# Baicalein Ameliorates *Streptococcus suis*-Induced Infection In Vitro and In Vivo

**DOI:** 10.3390/ijms22115829

**Published:** 2021-05-29

**Authors:** Hao Lu, Xiaodan Li, Gaoyan Wang, Chenchen Wang, Jiajia Feng, Wenjia Lu, Xiangru Wang, Huanchun Chen, Manli Liu, Chen Tan

**Affiliations:** 1 State Key Laboratory of Agricultural Microbiology, College of Veterinary Medicine, Huazhong Agricultural University, Wuhan 430000, China; 88251420@webmail.hzau.edu.cn (H.L.); xiaodanLi@webmail.hzau.edu.cn (X.L.); 97wgy@webmail.hzau.edu.cn (G.W.); 2018302110164@webmail.hzau.edu.cn (C.W.); 19fj@webmail.hzau.edu.cn (J.F.); 2017302110131@webmail.hzau.edu.cn (W.L.); wangxr228@mail.hzau.edu.cn (X.W.); chenhch@mail.hzau.edu.cn (H.C.); 2 Hubei Biopesticide Engineering Research Centre, Wuhan 430000, China; manli.liu@nberc.com; 3 Key Laboratory of Preventive Veterinary Medicine in Hubei Province, The Cooperative Innovation Center for Sustainable Pig Production, Wuhan 430000, China; 4 Key Laboratory of Development of Veterinary Diagnostic Products, Ministry of Agriculture of the People’s Republic of China, Wuhan 430000, China; 5 International Research Center for Animal Disease, Ministry of Science and Technology of the People’s Republic of China, Wuhan 430000, China

**Keywords:** baicalein, *Streptococcus suis*, antibacterial, anti-hemolysin, suilysin

## Abstract

As an important zoonotic pathogen, *Streptococcus suis* (*S. suis*) infection has been reported to be a causative agent for variety of diseases in humans and animals, especially Streptococcal toxic shock-like syndrome (STSLS), which is commonly seen in cases of severe *S. suis* infection. STSLS is often accompanied by excessive production of inflammatory cytokines, which is the main cause of death. This calls for development of new strategies to avert the damage caused by STSLS. In this study, we found for the first time that Baicalein, combined with ampicillin, effectively improved severe *S. suis* infection. Further experiments demonstrated that baicalein significantly inhibited the hemolytic activity of SLY by directly binding to SLY and destroying its secondary structure. Cell-based assays revealed that Baicalein did not exert toxic effects and conferred protection in *S. suis*-infected cells. Interestingly, compared with ampicillin alone, Baicalein combined with ampicillin resulted in a higher survival rate in mice severely infected with *S. suis*. At the same time, we found that baicalein can be combined with meropenem against MRSA. In conclusion, these results indicate that baicalein has a good application prospect.

## 1. Introduction

*Streptococcus suis* (*S. suis*) infection is associated with high mortality in humans and pigs. It induces symptoms, including septicaemia, meningitis, arthritis, and endocarditis. According to incomplete statistics, since the first case in 1968, there have been reports of more than 1600 human *S. suis* infections [1]. *S. suis* is transmitted through skin wounds or oral or nasal mucosa in persons who contact sick pigs, carry pigs, or raw pork [2]. Among the known serotypes of *S. suis*, serotype 2 (SS2) is the most prevalent in pigs and humans globally [3]. *S. suis* is similarly considered a lethal pathogen linked to meningitis in Vietnam, Thailand, and Hong Kong [4,5]. The survivors of *S. suis*-induced meningitis, in most cases, present with severe sequelae such as deafness [6]. SS2 is an emerging zoonotic pathogen that infects humans and pigs. It causes meningitis, endocarditis, and streptococcal toxic shock-like syndrome (STSLS) in humans [7]. In 2005, 215 cases of humans *S. suis* infection were reported in Sichuan province. Existing reports show that *S. suis*-derived STSLS causes acute high fever, hypotension, shock, blood spots and multiple organ dysfunction, and sometimes acute death. Collectively, *S. suis* infection poses a serious public health threat. Thus, elucidating the pathological factors associated with *S. suis* infection would guide the devising of effective strategies to minimize the associated social burden.

To infect the host, *S.* *suis* must successfully traverse epithelial barriers, evade the host’s immune system, multiply in the bloodstream, and invade various organs. Consequently, the necrosis of tissue cells or organs occurs [4,8]. Previous studies have implicated suilysin (SLY) as a crucial virulence factor that exerts a significant role in the pathogenesis of SS2 infection and induction of inflammatory response [9,10]. Mice infected with SS2 strain ST1 produce high levels of suilysin and show mortality rates close to 90% within 10 days. A previous study revealed that SS2 Strain ST104 produced low levels of suilysin and induced lower pathogenicity in mice [11]. Additional reports indicate that *S. suis* with high SLY levels are more likely associated with higher mortality in infected models than non-virulent strains [11,12]. Thus, it is suggested that the pathogenicity of *S. suis* is potentially enhanced by increasing SLY production. In addition, the NLRP3 inflammasome activation induced by SS2 strain SC19 is associated with elevated SLY production, which primarily justifies the excessive inflammatory response and multi-organ damage in STSLS [13]. SLY, a secreted protein, is a critical virulence factor for SS2 in the successful colonization of host cells and immune system evasion by the host. Therefore, an anti-virulent compound that effectively impedes the hemolytic suilysin activity could serve as a novel therapeutic agent for managing SS2 infection.

Inflammatory storms are among the foremost causes of death from numerous diseases such as the SARS-CoV epidemic in 2003 and the COVID-19 epidemic 2020, which severely progresses to cytokine storm syndrome, organ dysfunction, and death [14]. Previous studies also show that SS2 strain SC19 potentially secretes suilysin encoded by the SLY gene and is highly pathogenic to mice and pigs. It causes STSLS, characterized by the bacterial burden, inflammatory cytokine storm, multiple system organ failures, and acute death of the final host [15,16,17,18]. Of note, the degree of excessive inflammatory response and organ damage induced by SC19 is much higher than that of the classical virulent strain P1/7, potentially related to increased mortality [13]. Therefore, inhibiting *S. suis*-derived inflammatory cytokine storm is the key to curing streptococcicosis.

In other previous experiments, the virulence of SS2 was reduced by flavonoids, such as amentoflavone and fisetin, which could weaken the pathogenicity of SS2 by inhibiting the hemolytic activity of SLY. However, these compounds did not demonstrate satisfactory protection in animal experiments [15,19,20]. In the present study, we illustrate how baicalein binding to SLY protein active centers can effectively inhibit the hemolytic activity of suilysin. Baicalein can not only neutralize the toxicity of hemolysin, but also play a better bactericidal effect in combination with ampicillin. In particular, combined treatment with baicalein at a later stage of infection showed a higher survival rate of mice compared to when ampicillin is used alone. These findings implicate baicalein as a promising candidate for the treatment of *S. suis* infection.

## 2. Results

### 2.1. In Vitro Activity of Baicalein in Combination with Meropenem/Ampicillin against MRSA and Ampicillin-Resistant S. suis

Baicalein, alone or in combination with ampicillin/meropenem, was tested against reference strains and clinical isolates of MRSA. The MICs are shown in Table 1. Baicalein alone showed no obvious antibacterial effect (MIC > 128μg/mL). Baicalein at 16 μg/mL in combination with ampicillin/meropenem significantly increased the activity of medicine against all MRSA and *S. suis*. This concentration of baicalein was chosen based on data from checkerboard assays in which 16 μg/mL baicalein was the most common synergistic concentration with MRSA and *S. suis*.

### 2.2. Baicalein Inhibits the Hemolytic Activity of SLY without Altering the SS2 Growth

**Figure 1:**
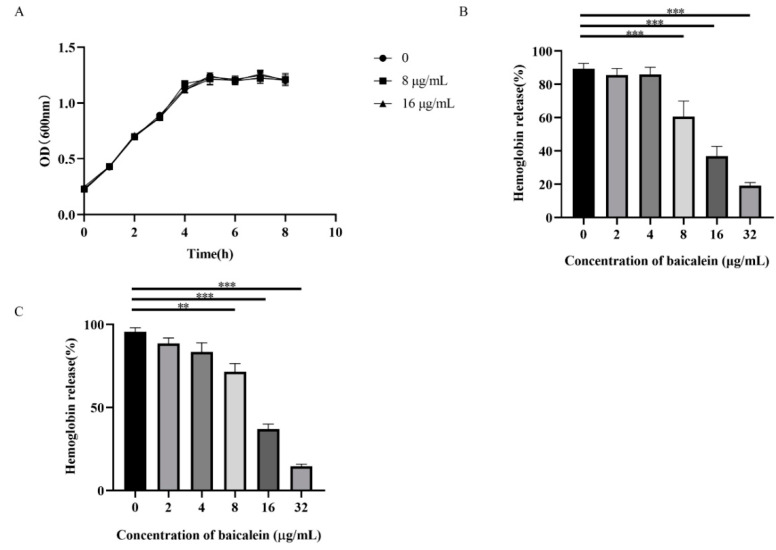
Baicalein inhibits the hemolytic activity of SLY: (**A**) The growth curve of SC19. SC19 was cultured with 5% newborn bovine serum and treated with different concentrations of baicalein; (**B**) Hemolytic activity of supernatants from SC19 and baicalein co-culture system; ** *p* < 0.01, *** *p* < 0.001 vs. 0 μg/mL. (**C**) Effect of baicalein on the hemolytic activity of purified SLY (100 ng/mL). ** *p* < 0.01, *** *p* < 0.001 vs. 0 μg/mL.

Using the growth curve, we validated whether the growth of SS2 was not significantly inhibited by following an increase in baicalein concentration from 8 to 16 μg/mL (Figure 1A). Notably, the supernatant of the SC19 medium showed hemolytic activity (Figure 1B). The hemolytic activity of the supernatant from the co-culture system of SC19 and baicalein (8 to 32 μg/mL) was significantly lower than that of SC19. We deduced that baicalein lowered the hemolytic activity of SC19 culture supernatant in a dose-dependent manner. The hemolytic activity of purified SLY decreased significantly with elevated baicalein concentration (0 to 32 μg/mL) (Figure 1C). Thus, it was evident that baicalein directly inhibited the hemolytic activity of SLY.

### 2.3. Baicalein Reduces SS2-Mediated Cytokine Production at the Cellular Level

**Figure 2:**
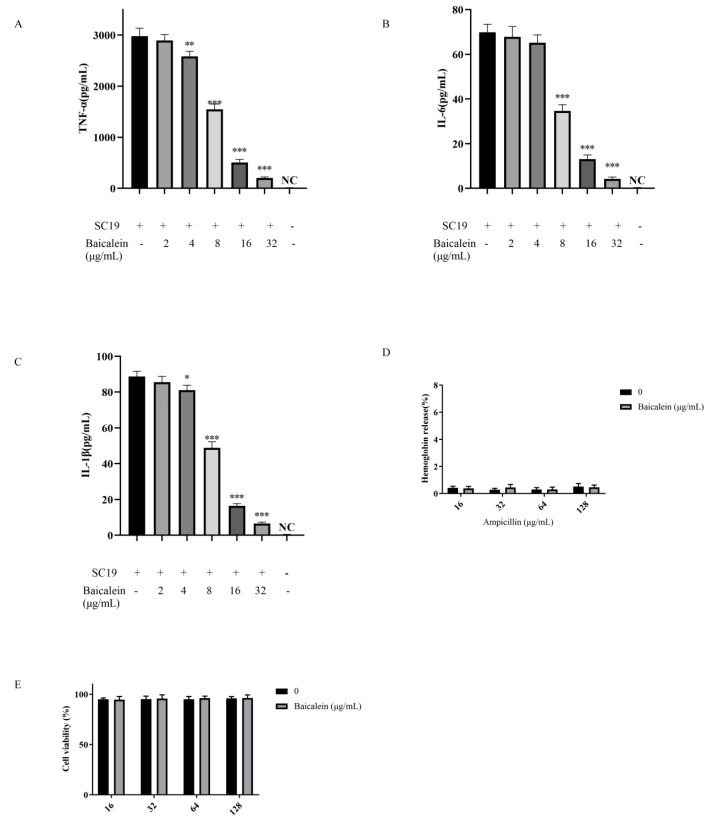
Baicalein reduced SS2-mediated cytokine production at the cellular level: (**A**) TNF-α; (**B**) IL-6, and (**C**) IL-1β. Cells were incubated with SS2 (MOI = 10:1) and different concentrations of baicalein for 6 h. ELISA was used to determine the concentrations of TNF-α, IL-1 β, and IL-6. “NC” represents no treatment with SC19. * *p* < 0.05, ** *p* < 0.01, *** *p* < 0.001 vs. SS2 alone; (**D**) Hemolytic activity of ampicillin to the RBCs in the absence or presence of baicalein; (**E**) The addition of baicalein exerts a negligible effect on the cytotoxicity of ampicillin in VERO cells.

*S. suis* infection potentially induces the host to produce several proinflammatory cytokines. Herein, STSLS was, in most cases, accompanied by excessive production of inflammatory cytokines, including TNF-α, IL-6, and IL-1β. To evaluate the immunomodulatory activity of Baicalein on *S. suis* infected macrophages, J774 cells were incubated with SS2 (multiplicity of infection [MOI] = 10:1) and various concentrations of Baicalein for 6 h. The levels of TNF-α, IL-6, and IL-1β were measured by enzyme-linked immunosorbent assays (ELISAs). The levels of TNF-α (Figure 2A), IL-1β (Figure 2B), and IL-6 (Figure 2C) in supernatants were significantly lower in SS2-infected cells treated with baicalein (2 to 32 μg/mL) than in untreated SS2-infected cells.

### 2.4. Safety Evaluation of Baicalein

In the clinic, the key problem of combination therapy is whether the toxicity of antibiotics together with adjuvants increases. After assessing the hemolysis and cytotoxicity of ampicillin to mammalian cells in the presence and absence of baicalein, the effect of high-level baicalein (32 μg /mL) on the hemolysis of erythrocytes (RBC) and cytotoxicity of Vero cells was negligible (Figure 2D,E).

### 2.5. Baicalein Alleviates SC19-Induced Injury of RAW264.7 Cells

**Figure 3:**
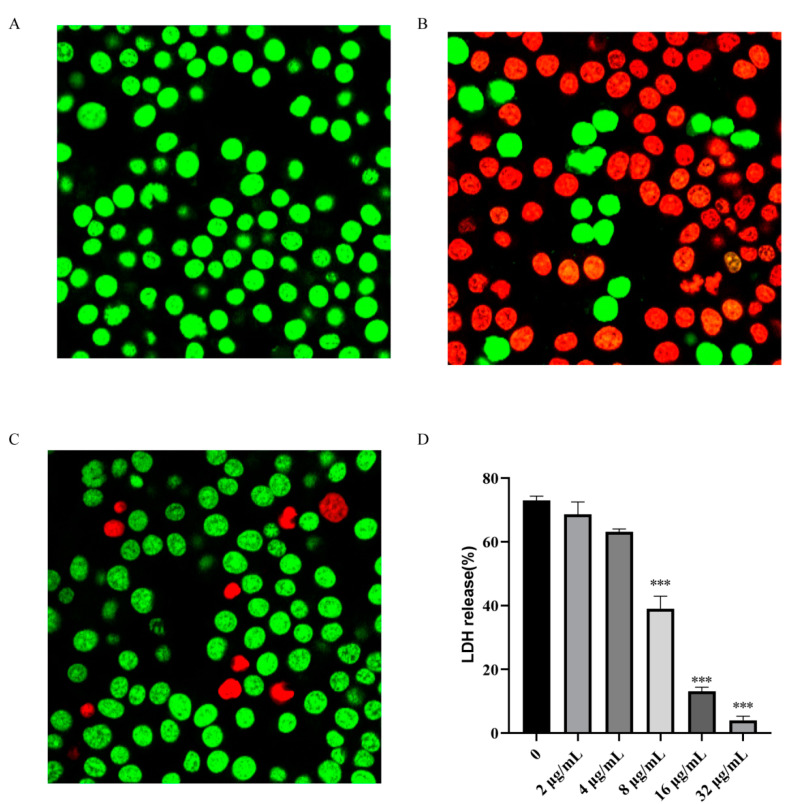
Baicalein protects J774 cells from SC19-mediated cell damage: (**A**) The cells not infected with SC19; Scale bar, 10 µm. (**B**) SC19-infected cells; (**C**) SC19-infected cells treated with 32 μg/mL baicalein; (**D**) LDH release from SC19-infected cells treated with different concentrations of baicalein (0 to 32 μg/mL). The results are presented as mean ± SD (*n* = 3). *** *p* < 0.001 vs. 0 μg/mL.

*S. suis* is not a typical intracellular bacterium. SLY, a virulence protein of Type 2 *S. suis*, mediates the invasion of *S. suis* into the host cell due to its perforation effect. *S. suis* can enter different cell types in mammalian tissues, such as macrophages, and evade host defenses, which prolongs the infection. This form of infection is highly challenging, because many antibiotics cannot penetrate the cell membrane and enter the intracellular niche to kill the bacteria. In this study, the uninfected RAW264.7 cells showed green fluorescence when stained with live/dead (green/red) staining reagent (Figure 3A). Following SC19 infection for 1 h, the amount of red fluorescence increased in RAW264.7 cells (Figure 3B), revealing that SC19 caused cell injury and death. Notably, the addition of baicalein 32 μg/mL protected RAW264.7 cells from SC19 mediated cell damage, which was verified by the significant reduction of red fluorescence (Figure 3C). LDH released into the supernatant of the co-culture system was measured by using a cytotoxicity test kit (LDH; Roche, Basel, Switzerland), and cell viability was determined [21]. It was found that baicalein inhibited LDH release in a dose-dependent manner. These results indicate that baicalein can inhibit the cell damage induced by SC19 (Figure 3D).

### 2.6. Identification of the Binding Sites between Baicalein and SLY

**Figure 4:**
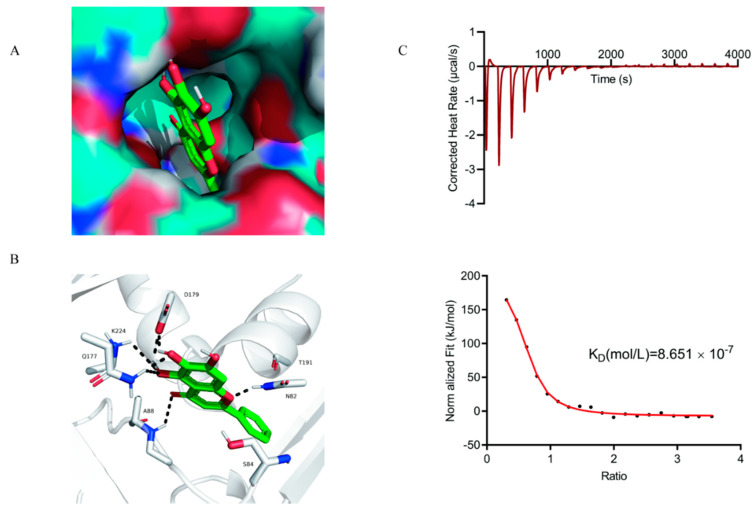
Baicalein destroys hemolytic activity based on the direct binding to the SLY; (**A**) Baicalein is docked into the binding site of the SLY (Total view); (**B**) Baicalein and SLY binding site (detailed view). The baicalein is represented with green sticks; the hydrogen bond is shown in a black dotted line; (**C**) ITC analysis of the interaction between SLY and baicalein; 0.5 mmol/L of baicalein was dropped into 0.02 mmol/L of SLY in PBS buffer at 25 °C. Calculated thermodynamic parameters, including the equilibrium dissociation constant (KD = 8.651 × 10^−7^ mol/ L).

We established the appropriate binding site of the baicalein in SLY using the molecular docking method. The estimated binding energy was −6.42 kcal/mol, then illustrated the hypothetical binding mode of the baicalein in the binding site of the SLY. In this model, baicalein adopted compact conformation to adhere to the binding site of the SLY (Figure 4A). Baicalein and protein amino acids, SER84 and THR191, formed strong hydrophobic interaction. Baicalein formed hydrogen bond interactions with amino acids ASN82, ALA88, GLN177 and ASP179, and LYS224. These interactions stabilized the protein and the compound baicalein (Figure 4B) and mediated anchorage of the baicalein in the binding site of the SLY. Isothermal titration calorimetry (ITC) was applied to establish the interaction of baicalein with SLY. From the assay results, the equilibrium dissociation constant (KD) between baicalein and SLY was 8.651 × 10^-7^ mol/L (Figure 4C), implying a high affinity to baicalein and SLY.

### 2.7. Baicalein Alters the Secondary Structure of SLY

We adopted CD spectroscopy to evaluate the effect of different baicalein concentrations on the conformation of SLY. The calculated secondary structure is outlined in Table 2. Baicalein induced a change in the conformation of SLY. After co-incubation of protein and baicalein, the percentage of SLY in the α-helical conformation decreased, whereas the proportions of β-sheets and β-turns increased. These results affirmed that baicalein caused the conformational changes of SLY molecules after acting on the protein.

### 2.8. Therapeutic Effects of Baicalein Combined with Ampicillin on SS2 SC19-Infected Mice

**Figure 5:**
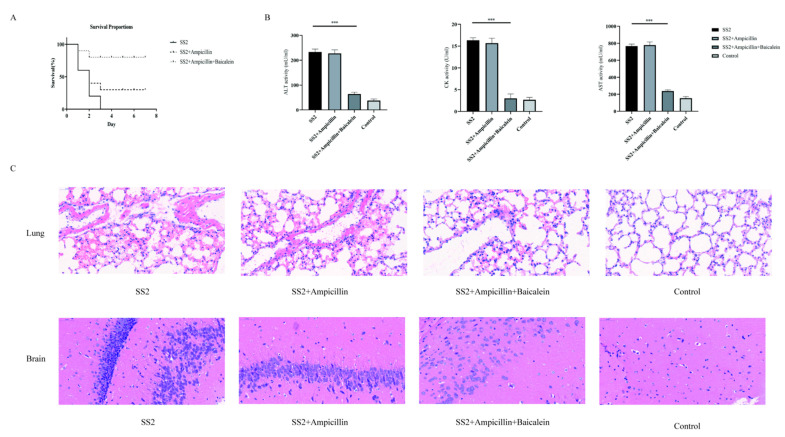
The survival rates and tissue pathological changes of SC19-infected mice; (**A**) The survival rates of baicalein + ampicillin and ampicillin cured severely infected mice model; (**B**) Blood levels of AST, ALT, and CK at 6 h post-infection (two-tailed, unpaired *t*-tests, *n* = 5); *** *p* < 0.001 vs SS2 alone. (**C**) Pathological changes of lung and brain tissue after baicalein and ampicillin treatment. Baicalein alleviated tissue damage of infected mice. The H&E images (40×) of tissue lesions.

The in vivo therapeutic effect of baicalein was evaluated using a mouse model severely infected with *S. suis* SC19. The protective effect of baicalein on infected mice was assessed based on the survival rate. The survival rate of baicalein combined with ampicillin-treated mice increased by 80% compared to that of untreated infected mice (Figure 5A), whereas that reported in the ampicillin-treated group was only 30%. Afterward, baicalein combined with ampicillin significantly relieved inflammation and pathological damages such as infiltration of inflammatory cells, alveolar interstitial congestion, and oedema in the lung and brain of infected mice (Figure 5C). Moreover, following the assessment of the effect of baicalein and ampicillin on the levels of alanine transaminase (ALT), aspartate transaminase (AST), and creatine kinase (CK), baicalein combined with ampicillin obviously decreased the levels of blood biochemistry in infected mice (Figure 5B). These data demonstrated that the anti-inflammatory effect of baicalein is essential in improving the survival of *S. suis* severe infected mice.

## 3. Discussion

With the elevated antibiotic resistance in many clinically relevant bacteria, there is an immediate need to develop new classes of antibacterial drugs that are not influenced by resistance mechanisms [22,23]. The mechanism of conventional antibiotics is to alter the basic functions of bacteria, for example, cell wall synthesis, DNA replication, or protein synthesis [24]. The development of drugs targeting virulence factors has become an important alternative to the management of drug-resistant bacterial-related infections [25]. As an essential virulence factor, SLY was shown to activate high levels of the inflammasome, which are crucial in STSLS [26]. SLY also plays a vital role in *S. suis*-induced meningitis [11]. Thus, targeting SLY related to inflammation is a novel strategy for *S. suis* treatment and relieves the development of antibiotic-resistant *S. suis*. Antiviral factors are superior to traditional antibiotics in two key aspects. First, they suppress the target genes necessary for basic metabolism. These genes are critically important in the pathogenic process and allow bacterial proliferation in the host. Second, drugs targeting virulence factors exert specific protective effects against bacteria in normal flora [25].

In this study, baicalein significantly reduced the hemolytic activity of SC19 culture supernatants and the purified recombinant SLY. Meanwhile, baicalein demonstrated no effect on the growth of *S. suis* SC19 at a concentration that effectively inhibited the hemolytic activity of SLY. Collectively, baicalein exerts less selective pressure for survival than conventional antibacterial agents in treating SS2 infection. Circular dichroism (CD) spectroscopy is an excellent method for assessing the structural changes of proteins under varying conditions [27]. CD spectrum was applied to assess the secondary structure of SLY post baicalein treatment. The change in secondary structure is related to the interaction between the protein and other components [28]. The molecular docking results suggested that one potential binding site existed in protein SLY and interacted with baicalein via hydrophobic interactions and hydrogen bonds. Moreover, the KD values of the ITC assay revealed that baicalein could interrupt protein-receptor interaction via strong direct binding to the SLY in vitro; this is the main reason for the anti-hemolysin activity of baicalein. We found that baicalein could directly target SLY to exert its antiviral effect but did not exert selective pressure on SS2 for survival. This implicates baicalein as a promising effective candidate for the treatment of *S. suis*-related infections. To prove this phenomenon, some verifications were subsequently undertaken at the animal model and cell level. Consistent with the above expectation, baicalein treatment significantly alleviated the cell damage to SS2 infection under concentrations that do not influence the growth of SS2. Although traditional antibiotics can effectively eliminate *S. suis*, these antibiotics cannot improve the survival rate of infected mice via the inhibition of the excessive proinflammatory responses [29]. Additionally, we revealed that the adoption of the first-line drug ampicillin in cases of nonresistant *S. suis* to a mouse model of severe infection maintained low protection rates (30%). However, after the addition of baicalein, there was a significant improvement in survival (80%). The present findings demonstrate that baicalein neutralizes hemolysin toxicity and reduces the inflammatory response, and is the primary cause of this phenomenon. Thus, for some severely infected patients, baicalein in combination with β-lactams drugs is potentially a better choice.

Compelling evidence shows that inflammation plays a significant role in protecting the human body from infection by various pathogens. However, excessive inflammation is unsuitable for the body and may cause lethal diseases [26]. Excessive inflammation is potentially associated with organ damage and accelerates disease progression, which a serious consequence of *S. suis* infection. The severity of SS2 infection is highly associated with the host’s natural immune response. The host immune system can release numerous proinflammatory cytokines following SS2 stimulation, including TNF-α, gamma interferon (IFN-γ), IL-1β, and IL-6 [18,30]. Additionally, excessive inflammation is related to some clinical symptoms of SS2 infection, including meningitis, sepsis, septic shock, and sudden death [31]. Therefore, alleviating excessive inflammation is one critical approach to managing the consequences of SS2 infection. Upon exploring the effect of baicalein on the anti-inflammatory activity of SS2 infection, baicalein significantly inhibited the production of TNF-α, IL-1β, and IL-6 in the supernatant of J774 cells infected with SS2 in a dose-dependent manner. In addition, baicalein, in combination with the first-line drug ampicillin treatment, lowered the levels of blood biochemistry (ALT, AST, CK) and bacterial load in the tissues of the mice infected with SS2 strain SC19. In consequence, we reported a higher survival rate of infected mice compared to that of infected mice treated with the single anti-hemolysin compound fisetin [19]. A critical concern for combinational therapy in the clinic is whether there is increased toxicity of antibiotics together with adjuvants. We revealed that baicalein in combination with ampicillin did not increase blood and cell toxicity. In summary, these findings demonstrate that baicalein is a potential novel therapeutic approach to managing S. suis infection due to its anti-hemolysin activity. In addition, the antibacterial test and survival rate test also proved that baicalein could cooperate with meropenem to resist MRSA infection, which needs further study. Thus, this study lays a foundation for sintering baicalein into a new potential drug.

## 4. Materials and Methods

### 4.1. Bacterial Strains, Growth Conditions, Baicalein Preparation

SS2 strain SC19 was isolated from the brains of dead pigs during the *S. suis* outbreak in Sichuan Province in 2005 [8]. Five clinical multidrug-resistant *S. suis* strains were obtained from our laboratory strains library and five MRSA strains were separated from Heze municipal hospital. The bacterial strains were cultured in Tryptose Soya broth (TSB) or plated on Tryptose Soya agar (TSA) (Summus Ltd.,Shanghai China) with 5% (*v*/*v*) newborn bovine serum (Sijiqing Ltd.,Shanghai China) at 37 °C. Baicalein was acquired from Topscience. We used dimethyl sulfoxide (DMSO, Sigma-Aldrich, St. Louis, MO USA) to dissolve the drugs.

### 4.2. Antibacterial Tests

The determination of the minimum inhibitory concentration of compound referred to the Clinical and Laboratory Standards Institute (CLSI) guideline [32]. Microdilution broth method was applied in 96-well plates (Corning Costar^®^ 3599, Corning, NY, USA) using MH. The final concentration of the culture was 1.5 × 10^6^ colony-forming units (CFU)/mL. After 18 h incubation at 37 °C, the MICs were defined as the lowest concentrations of antibiotics with no visible growth of bacteria. The measurement was repeated in triplicate.

### 4.3. Baicalein on the Growth Assay of SC19

SC19 overnight culture containing 5% newborn bovine serum in TSB was diluted into 10 mL equal parts to a final concentration of 5 × 10^5^ CFU/mL. Then, varying baicalein concentrations (0, 8, and 16 μg/mL) were added to the cell culture plate. To assess the effect of baicalein on SC19, an automatic microbial growth curve analysis system (Bioscreen C) was employed to examine bacterial growth at an optical density of 600 nm (OD600) every 30 min [8].

### 4.4. Preparation of Recombinant SLY Protein and Anti-SLY Protein Hemolysis Assay

To construct the prokaryotic expression plasmid pET-28a(+)-SLY, we subcloned SLY cDNA to pET-28a(+) vector (Novagen, Madison, WI, USA) using BamHI and NdeI restriction enzyme cutting sites. *E. coli* BL21 transformed with the recombinant plasmid was cultured in a medium containing 0.2 mM isopropyl-β-d-thiogalactopyranoside (IPTG), then induced for 16 h at 16 °C. Purification of the resultant SLY was achieved by loading the supernatant of bacterial cell lysates onto a Ni-NTA column. The antihemolytic activity of baicalein was directly evaluated through co-incubation with the purified protein (100 ng/mL) and baicalein at different concentrations (0, 2, 4, 8, 16, and 32 ug/mL) following the above-described protocol.

### 4.5. Evaluating Baicalein Activity against the Hemolytic Activity of SLY

*S. suis* strain SC19 was cultured for 12 h at 37 °C. The cultures were centrifuged at (12,000 rpm, 15 min) 4 °C. The collected supernatant was incubated for 30 min with the final concentrations of baicalein as follows: 0, 2, 4, 8, 16, and 32 ug/mL, at 37 °C. This was followed by the addition of 2% defibrated red sheep blood and a 30-min incubation at 37 °C. Finally, the mixture was centrifuged at 1000 rpm for 5 min at 4 °C. Aliquots of the supernatant (200 uL) were obtained via a BioSpectrometer (FLUOstar Omega, Offenburg, Germany) at an optical density of 543 nm. The sample was treated with 2.5% TritonX-100 as a 100% cleavage control. For each sample, the ratio of OD543 to the 100% cleavage control activity of baicalein was evaluated against the hemolytic activity of SLY.

### 4.6. Safety Assessment

Here, 2% of defibrillated sheep red blood cells were incubated with ampicillin (16–128 μg/mL) or baicalein (32 μg/mL) at 37 °C for 1 h. Phosphate buffer (PBS, pH = 7.4) served as a positive control and negative control with or without 2.5% Triton X-100, respectively. Fluostar Omega was applied to evaluate the absorption of released hemoglobin at 543 nm. The following formula was used to assess the hemolysis rate: Hemolysis (%) = [(OD540 sample − OD540 blank)/(OD540 2.5% Triton X-100 − OD540 blank)] × 100%. The CCK-8 (US Everbright^®^ Inc., Suzhou, China) assay was employed to assess the cytotoxicity on VERO cells at 450 nm absorbance. Ampicillin (16–128 µg/mL) with baicalein (32 μg/mL) and 1 × 10^4^ cells were added to 96 well plates simultaneously and cultured in DMEM with 10% heat-inactivated FBS at 37 °C for 24 h. After that, CCK-8 (US Everbright^®^ Inc., Suzhou, China) was added.

### 4.7. Circular Dichroism Analysis

We co-incubated baicalein with purified SLY (0.5 μg/mL) at 37 °C for 1 h. A CD spectrophotometer (MOS-500; Bio-Logic, grenoble France) was applied to establish the secondary structure of SLY at room temperature (25 °C). The scanning wavelengths and rate were 190 to 250 nm and 50 nm/min, respectively. The bandwidth was 1.0 nm. Bestsel webserver was used to analyze the secondary structure of SLY [33].

### 4.8. Cell Culture and Infection

Mouse macrophage-like cells RAW264.7 were cultured at 37 °C in 5% CO_2_ in DMEM (Invitrogen, Carlsbad, CA, USA.) with 10% fetal bovine serum (FBS). The cells (density of 2 × 10^4^ cells per well) were inoculated overnight on 96-well plates, then infected with SC19 at OD600 = 0.8 and resuspended in FBS-free DMEM medium at MOI = 10 for 1 h. After washing three times with PBS, fresh DMEM containing concentrations of baicalein (0–32 μg/mL) was added and left for 6 h. RAW264.7 cells treated with DMEM with or without 2.5% Triton X-100 served as positive and negative controls. The supernatant was collected from 96-well plates via centrifugation (400 rpm, 5 min). LDH released into the supernatant was evaluated using an LDH cytotoxicity assay kit (c0016, Beyotime, Shanghai China). Microscopic images of stained cells (Baicalein 32 μg/mL) were obtained using live/dead (green/red) reagents (Invitrogen) under a confocal laser scanning microscope (Nikon, Tokyo, Japan).

### 4.9. Homology Modeling and Molecular Docking

The search for the amino acid sequence of the *S. suis* SLY was conducted in the NCBI protein database (http://www.ncbi.nlm.nih.gov/protein/). The protein sequence was NC_012924.1. We employed Autodock Vina 1.5.6 for the molecular docking study of SLY and baicalein; the new scoring function improved the docking speed and accuracy. Using ChemBioDraw Ultra 14.0 and ChemBio3D Ultra 14.0, the 2D and 3D structures of baicalein were drawn. The docking input file was generated via Autodock Tools 1.5.6 [34,35].

### 4.10. Isothermal Titration Calorimetry (ITC) Assay

The interaction of the SLY protein with baicalein was assessed via calorimetry using affinity ITC (TA NANO ITC) in vitro. The purified SLY protein (0.02 mmol/L) and baicalein (0.5 mmol/L) were dissolved in PBS (pH = 7.4). Baicalein was injected into a sample cell filled with the purified SLY protein. The injection was repeated 20 times with an equilibrium interval of 200 s. This experiment was performed at 25 °C. The nanoAnalyzer software was employed to generate an equilibrium dissociation constant (KD).

### 4.11. Establishing an SS2 SC19-Infected Mouse Model In Vivo

Seven-week-old female BALB/c mice were purchased from China Three Gorges University and used to establish a mouse model of SS2 severe SC19 infection. Animal experiments conformed to animal ethics. All experiments strictly adhered to the guidance of the Protection, Supervision, and Control Committee of Animal Experiments of Huazhong Agricultural University (HZAUMO-2021-0008). SS2 SC19 was transferred into TSB medium at 1:100 and cultured at 37 °C until OD600 = 0.6. The bacteria were collected via 10-min centrifugation (10,000 rpm at 4 °C) and then suspended with PBS (pH = 7.4). For the survival rate assay, the SC19 concentration in intraperitoneally infected mice was 2.5 × 10^9^ cells/mL (200 µL). After a six-hour infection, mice were treated with baicalein (5 mg/kg) and ampicillin (5 mg/kg) or ampicillin (5 mg/kg) alone via intraperitoneal injection. The interval of each treatment was 12 h. The control group (10 per group) was injected with an equal volume of PBS (pH = 7.4). The survival curve of mice was generated based on assay data.

Moreover, mice (5 per group) were intraperitoneally infected with SC19 (200 µL) at a concentration of 2.5 × 10^8^ cells/mL. Then, baicalein or ampicillin was injected via the same protocol described above. The control group received PBS (pH = 7.4). At 12 h post-injection, the cardiac blood of anesthetized mice was collected and used to assess the effects of baicalein on the levels of blood biochemistry (ALT, AST, CK) in SC19-infected mice. Finally, the pathological changes of lung and brain tissue were explored following immobilization in 4% paraformaldehyde.

## 5. Conclusions

The SLY protein causes a cytokine storm associated with increased morbidity and mortality in *S. suis* infection. The present study demonstrates that baicalein can relieve *S. suis* infections by targeting SLY and inhibiting inflammation. Additionally, the combination of Baicalein with ampicillin is linked to a good therapeutic effect; thus, combined use of anti-virulence drugs with clinical antibacterial drugs can be applied to treat severe *S. suis* infections. The antibacterial test also proved that baicalein could cooperate with meropenem to resist MRSA infection. In a nutshell, these findings implicate baicalein as a promising therapeutic candidate for *S. suis* and MRSA infection. Meanwhile, it is suggested that baicalein is particularly important to strengthen the monitoring of inflammatory response and to treat bacterial infections that are associated with a severe inflammatory response.

## Figures and Tables

**Table 1:** Minimum inhibitory concentrations (MICs) of baicalein (Ba) and ampicillin (Am)/meropenem (Me), alone or in combination, against *S. aureus* and MRSA.

Species	Isolate	MIC (mg/L)				Fold Change in MIC of Antibiotic
		Ba Alone	Am Alone	Me Alone	Antibiotic in Combination ^a^	
*S. suis*	S21	>128	16		0.5	32
	S18	>128	32		1	32
	S26	>128	32		0.5	64
	S15	>128	16		0.5	32
	S32	>128	64		2	32
MRSA	M1002	>128		16	0.5	32
	M1015	>128		8	0.125	64
	M1025	>128		16	0.5	32
	M1011	>128		32	1	32
	M1032	>128		64	1	64

^a^ MIC of antibiotic in combination with 16 μg/mL Ba.

**Table 2:** Determination of secondary structure components of sly treated with apigenin at different concentrations.

Concentration of Baicalein (μg/mL)	Content (%)				NRMSD ^a^
	α-Helix	β-Sheet	β-Turns	Others	
0 (control)	15.1	39.6	12.4	32.9	0.03756
32	1.3	44.5	18.2	36	0.06547

^a^ NRMSD, normalized root-mean-square deviation.

## Data Availability

The data presented in this study are available on request from the corresponding author.
